# Accelerated Development of the First-Order Central Auditory Neurons With Spontaneous Activity

**DOI:** 10.3389/fnmol.2018.00183

**Published:** 2018-05-31

**Authors:** Xin-Lu Yin, Hui-Qun Jie, Min Liang, Li-Na Gong, Han-Wei Liu, Hao-Lai Pan, Ya-Zhi Xing, Hai-Bo Shi, Chun-Yan Li, Lu-Yang Wang, Shan-Kai Yin

**Affiliations:** ^1^Department of Otorhinolaryngology, The Sixth People’s Hospital of Shanghai, Shanghai Jiao Tong University, Shanghai, China; ^2^Programs in Neurosciences & Mental Health, Department of Physiology, Sick Kids Research Institute, Toronto, ON, Canada

**Keywords:** spontaneous activity, developing neuronal networks, patch-clamp, HCN channels, synaptic transmission, synaptic pruning

## Abstract

In developing sensory systems, elaborate morphological connectivity between peripheral cells and first-order central neurons emerges via genetic programming before the onset of sensory activities. However, how the first-order central neurons acquire the capacity to interface with peripheral cells remains elusive. By making patch-clamp recordings from mouse brainstem slices, we found that a subset of neurons in the cochlear nuclei, the first central station to receive peripheral acoustic impulses, exhibits spontaneous firings (SFs) as early as at birth, and the fraction of such neurons increases during the prehearing period. SFs are reduced but not eliminated by a cocktail of blockers for excitatory and inhibitory synaptic inputs, implicating the involvement of intrinsic pacemaker channels. Furthermore, we demonstrate that these intrinsic firings (IFs) are largely driven by hyperpolarization- and cyclic nucleotide-gated channel (HCN) mediated currents (I_h_), as evidenced by their attenuation in the presence of HCN blockers or in neurons from HCN1 knockout mice. Interestingly, genetic deletion of HCN1 cannot be fully compensated by other pacemaker conductances and precludes age-dependent up regulation in the fraction of spontaneous active neurons and their firing rate. Surprisingly, neurons with SFs show accelerated development in excitability, spike waveform and firing pattern as well as synaptic pruning towards mature phenotypes compared to those without SFs. Our results imply that SFs of the first-order central neurons may reciprocally promote their wiring and firing with peripheral inputs, potentially enabling the correlated activity and crosstalk between the developing brain and external environment.

## Introduction

Spontaneous neuronal activity is a characteristic feature in the majority of developing sensory systems, such as the spinal cord, cerebellum, auditory and visual systems (Mohajerani and Cherubini, 2005; Kirkby et al., 2013; Del Rio-Bermudez et al., 2016). Even at an extremely early stage before the onset of sensory experience, neurons and neuronal precursors exhibit spontaneous electrical activity. This is thought to be crucial for regulation of neuronal survival and migration, refinement of synaptic connections, formation of neuronal maps and establishment of the precise circuitry required in adult nervous systems. The developing auditory system exemplifies a sensory activity-independent process (Kennedy, 2012), where by mice and rats do not hear until the external auditory canal has opened at postnatal (P) day 10–12 (when airborne sound is only able to elicit neural responses in the inner ear after this opening (Beutner and Moser, 2001; Glowatzki and Fuchs, 2002)). Indeed, much of the complex circuitry that underpins hearing has been established before the cochlea can reliably respond to sound and is therefore independent of sound evoked activity (Kros et al., 1998; Friauf and Lohmann, 1999), indicating that other mechanisms are engaged in guiding the establishment of the neural network.

Before the onset of hearing, developing inner hair cells (IHCs) are not mere quiescent cells, and can generate electrical activity in the form of calcium-based action potentials (Marcotti et al., 2003; Tritsch et al., 2010). Tritsch et al. (2007) discovered that supporting cells within Kölliker’s organ spontaneously release ATP to depolarize nearby IHCs and evoke glutamate release, driving bursts of firing activity in spiral ganglion neurons (SGNs) and propagation of spikes via auditory nerves into downstream nuclei (Tritsch et al., 2007). These compelling studies led to the prevailing view that spontaneous activity in the periphery plays an instructive role in the development of the central auditory brainstem prior to external sensory-evoked activity. However, it remains unknown whether the first-order central auditory neurons are capable of spontaneous discharge without peripheral inputs during the early postnatal stage; and if so, what the origin and molecular substrates underlying such activity are.

In this study, we investigated the electrophysiological properties of principal neurons in the cochlear nucleus (CN), the first central station innervated by auditory nerves, which plays a crucial role in processing and dispersing incoming acoustic information (Terreros and Delano, 2015; Nothwang, 2016). Surprisingly, we discovered that spontaneous firings (SFs) are present as early as P0–P1, when the auditory circuitry is not yet fully established. After blocking excitatory and inhibitory synaptic transmissions, SFs remain present, suggesting these activities are intrinsically driven. By combining pharmacological and genetic knockout approaches, we revealed that currents mediated by hyperpolarization- and cyclic nucleotide-gated channels (I_h_, HCN) dominate over other pacemaker channels in generating intrinsic firings (IFs). Furthermore, we demonstrate that this subset of neurons with IFs is uniquely associated with accelerated development in excitability and connectivity. Our results suggest that IFs in the first-order central neurons can potentially contribute to rapid maturation of sensory systems of the developing brain.

## Materials and Methods

All experimental protocols complied with the guiding principles for the care and use of animals and were approved by the Ethics Review Committee for Animal Experimentation at Shanghai Jiao Tong University. Both the number of animals used and their suffering were reduced as much as possible. HCN1 knockout mice were purchased from the Jackson Lab and bred in heterozygotes to produce age-matched WT and HCN1^−/−^ littermates for experiments.

### Preparation of Brain Slices

C57Bl/6J mice between postnatal day 0 and 19 were first anesthetized with sodium pentobarbital (55 mg/kg, i.p.) and then decapitated. The brains were quickly but carefully removed into ice-cold oxygenated (95% O_2_ and 5% CO_2_) incubation solution, dissected, and sectioned in transverse plane at a thickness of 300 μm using a micro slicer (VT-1000S, Leica Microsystems, Nussloch, Germany). Slices containing the cochlear nuclei were allowed to recover at 37°C for at least 1 h and then transferred to a recording chamber at room temperature before use.

### Solutions

The solution for the slice preparation contained (in mM): 124 NaCl, 5 KCl, 1.2 KH_2_PO_4_, 1.3 MgSO_4_, 2.4 CaCl_2_, 24 NaHCO_3_, and 10 glucose saturated with 95% O_2_ and 5%CO_2_. Patch electrodes were filled with the following solutions (in mM) for cell-attached and whole-cell recordings: 97.5 K-gluconate, 32.5 KCl, 0.5 ethylene glycol tetra acetic acid (EGTA), 404-(2-hydroxyethyl)-1-piperazineethanesulfonic acid (HEPES), and 1 MgCl_2_. Recording solution was identical to dissection solution though they were used at different temperatures. The cAMP (100 μM) was added to the above pipette solution, and HCN current was recorded 2 min after the establishment of whole cell configuration. The internal solutions were adjusted to a pH of 7.2 and 300 mMOsm.

### Electrophysiology

All recordings were made with the cell attach and whole cell patch-clamp technique using a patch-clamp amplifier (EPC-10; HEKA, Germany), and all data were sampled at 10–12 kHz and filtered at 1–3 kHz using a Dell computer equipped with Patch master (HEKA; 2.9 kHz Bessel filter, sampled at 10 kHz). The patch electrodes were pulled from borosilicate capillary glass pipettes by a vertical pipette puller (P-9; Narishige, Tokyo, Japan) and had a resistance of 3–8 MΩ when pipettes were filled with the pipette solution. To record spontaneous action potential currents, neurons were voltage-clamped at a holding potential of −60 mV or current-clamped at 0 pA to ensure data were recorded without any stimulation. For a majority of cell-attached recordings of spike firings, the holding potential was −60 mV for voltage-clamp mode. Tight seal (up to GΩ) ensures high signal/noise ratio, and whenever partial membrane ruptures spontaneously, the firing rate would increase abruptly. Under such circumstance, we would switch to current-clamp mode to register spontaneous firing without injecting any holding current. In control experiments, we compared the effects of holding potentials on firing rates in 15 spontaneously active neurons in voltage-clamp mode and did not observe any changes in the firing rate as had been reported previously (Alcami et al., 2012). This is likely due to the fact that the spike initiation site (e.g., AIS) is distal from the recording site and AP currents recorded in our experiments are the capacitive currents of back-propagated spikes. Similar firing rates and patterns in voltage- or current-clamp recordings from the same neurons validate the reliability of recording methods.

Electrode capacitance and liquid junction potential were compensated for and all experiments for whole-cell voltage-clamp recordings of I_h_ and EPSCs were done after approximately 90% online correction of the series resistance (5–10 MΩ) with proper membrane capacitance neutralization. Whole-cell current-clamp recordings were done after bridge balance. All experiments were performed at room temperature (21–26°C). To measure evoked EPSCs, a bipolar stimulation electrode was positioned to the auditory nerve stub to stimulate afferent inputs to the recorded cell. Recordings were made 200–400 μm away from the stimulation electrode. The threshold to evoke EPSCs was first measured by varying the stimulation intensity from 0 V to 20 V. For stellate cells, the stimulus delivered to the afferents was adjusted to be 1.5–2 times larger than the threshold in order to elicit reliable EPSCs. For bushy neurons in VCN, the intensity of the stimuli increased gradually to observe stepwise changes in eEPSCs. Paired-pulses stimulations with an inter-pulse time interval of 20 ms were repeated 3–4 times with an inter-trial interval of 15 s for any given intensity.

### Drugs

The drugs used in the experiments are in below table.

**Table:** 

Name of drugs	Concentration
Bicu (bicuculline)	10 μM
NBQX (2,3-dihydroxy-6-nitro-7-sulfamoyl-benzoquinoxaline-2,3-dione)	2 μM
Stry	1 μM
APV (DL-l-2-amino-5-phosphonovaleric acid)	50 μM
CsCl	2 mM
ZD7288	40 μM
A-803467	10 μM
8-Br-cAMP (8-bromo-adenosine-3′,5′-cyclicmonophosphate)	0.1 mM
SQ225, 36 (9-(Tetrahydro-2-furanyl)-9H-purin-6-amine)	50 μM
GTX1–15	0.01 μM
ODQ (1H-oxadiazolo[4,3-a]quinoxalin-1-one)	30 μM
DMSO (dimethylsulfoxide)	<0.1%

Chemicals and drugs were purchased from Sigma (St. Louis, MO, USA), except GTX1–15 which was purchased from Alomone Labs (Jerusalem, Israel). Bicu, CsCl and8-Br-cAMP were prepared in distilled water, while the other chemicals were dissolved in dimethylsulfoxide (DMSO; Sigma), and diluted to the required concentration in ACSF immediately prior to use, resulting in a maximal DMSO concentration of <0.1%. Application of drugs was achieved by switching a multi-valve, single-output gravity perfusion system made of square glass capillary (0.4 mm in width, Cat.#64–0121, Warner, USA) to the experimental chamber at a speed about 2 ml/min.

### Data Analysis

All data were stored on a personal computer for further analysis. Electrophysiological data were analyzed off-line using the Mini Analysis Program (Synaptosoft, Leonia, NJ, USA) and Clamp fit 10.2 software (Molecular Devices). Origin 8 (Microcal Software) and SigmaPlot12 (Systat Software) were used for graphic representation. The average values of the spontaneous firing frequencies during the control period were calculated and scaled to 1.0. All subsequent recordings during this type of experiment were normalized to the control value. Differences in the frequency of spontaneous activities were examined using Wilcoxon signed-ranks tests for comparison between groups. The percentages of intrinsic firing neurons in WT and HCN1KO were examined using Chi-squared test. Statistical analyses were performed using SPSS 17.0 software (SPSS Inc.). All numerical results are presented as means ± standard errors. *p*-values were considered to be statistically significant at different levels with different number of asterisk symbols (*p < 0.05, **p < 0.01, ***p < 0.001).

## Results

### Spontaneous Firings Exist in Neonatal CN Neurons and Are Developmentally Upregulated

**Figure 1:**
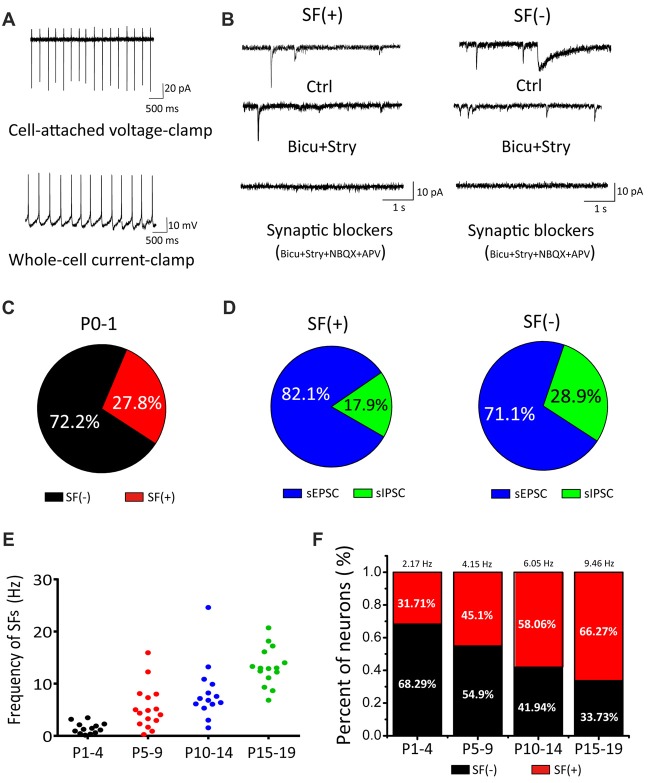
Cochlear nucleus (CN) neurons exhibited spontaneous firings (SFs) in the early postnatal stage. **(A)** Examples showing cell-attached recordings of SFs in voltage-clamp or current-clamp mode. **(B)** Typical spontaneous synaptic currents recorded in three sequential conditions (control, bicu+stry, bicu+stry+NBQX+APV) in SF(+) and SF(−) neurons. **(C)** Pie charts showing the percentage of SF(+) and SF(−) neurons. **(D)** The proportion of spontaneous inhibitory postsynaptic currents (sIPSCs) and spontaneous excitatory postsynaptic currents (sEPSCs) detected in SF(+) and SF(−) neurons, respectively. **(E)** A scattered dot plot showing developmental-dependent changes in the firing rate of 58 SF(+) CN neurons from P1–4, P5–10, P11–14 and P15–19 mice. **(F)** A bar graph summarizing the percentages of SF(+) and SF(−) neurons for four age groups with the mean firing rate for SF(+) neurons given (P1–4: 2.17 Hz, 32%; P5–10: 4.15 Hz, 45%; P11–14: 6.05 Hz, 58%; P15–19: 9.46 Hz, 66%).

In the auditory brainstem, the SGNs convey signals from IHCs to the principal neurons via glutamatergic inputs (i.e., auditory nerve) to the CN where incoming information is processed and dispersed for computation and coding in other central nuclei. To systematically study the properties of these first-order auditory neurons, we first acquired cell-attached recording of SFs in voltage-clamp or current-clamp mode (Figure 1A) from brainstem slices taken from postnatal mice at ages ranging from P0 to P19. A majority of recordings were performed randomly in cell-attached configuration to minimize perturbation to the intracellular homeostasis of neonatal CN neurons before cell-type specific signatures of different neurons could be reliably measured via electrophysiology. Using these experimental paradigms, we found that a fraction of CN neurons already exhibited SFs as early as P0–1 (Figure 1C; neurons with or without SFs are henceforth designated as SF(+) and SF(−), being 27.8% (*n* = 15) and 72.2% (*n* = 39), respectively, despite the fact that the presumed origin of upstream spontaneous activity (i.e., auditory nerve) had been cut during slice preparation). This is surprising in the context of compelling evidence showing that spontaneous activity in IHCs during the prehearing stage drives downstream propagation of signals (Tritsch and Bergles, 2010). Given that auditory nerve endings from SGNs remain attached in the slice preparation and potentially discharge to evoke postsynaptic firings, we subsequently performed whole-cell voltage-clamp recordings of spontaneous excitatory and inhibitory postsynaptic currents (sEPSCs and sIPSCs; i.e., inhibitory inputs from interneurons being potentially excitatory due to high intracellular Cl^−^ concentration in developing neurons during the early development). Figure 1B shows example traces of spontaneous postsynaptic currents recorded from SF(+) or SF(−) neurons at P0, which were identified and classified during cell-attached configuration. In both cases, we found a mixture of sIPSCs and sEPSCs with the former showing a much slower time course than the latter. These spontaneous synaptic events can be sequentially blocked by GABA/Glycine receptor antagonists bicuculine (bicu, 10 μM) and strychnine (stry, 0.3 μM), and NMDA/AMPA receptor antagonists APV (50 μM) and NBQX (2 μM). In no cases of recordings from P0–1 cells were spontaneous synaptic events absent. This observation confirms that functional synaptic connectivity between CN neurons and peripheral projections as well as local inhibitory inputs has indeed been achieved in the embryonic stage (Marrs and Spirou, 2012; Yu and Goodrich, 2014). Figure 1D shows the proportion of sEPSC/sIPSC in two subsets of neurons (SF(+): sEPSC: 82.1%, sIPSC: 17.9%, *n* = 5, *p* < 0.05; SF(−): sEPSC: 71.1%, sIPSC: 28.9%, *n* = 4, *p* < 0.05).

To explore the age-dependent changes in SFs, we recorded from CN neurons with a broad coverage of postnatal stages up to P19 when these neurons approach maturity. In this set of experiments, we made recordings from a total of 58 neurons and analyzed the fraction and mean frequency of SFs in four age groups (P1–4, P5–10, P11–14 and P15–19). We found both the fraction of SF(+) neurons and their discharge frequency increased with age. As summarized in Figures 1E,F, the average frequencies of the four groups were 2.17 Hz, 4.15 Hz, 6.05 Hz and 9.46 Hz (*n* = 13, 23, 18, 19, respectively) whereas the fraction of SF(+) neurons gradually increased across the same four groups, 31.71%, 45.1%, 58.06% and 66.27%, respectively. These results demonstrated that SFs are present throughout the entire postnatal development of principal neurons in the first central station of the auditory brainstem.

### SFs Are Intrinsically Driven and Augmented by Synaptic Inputs

**Figure 2:**
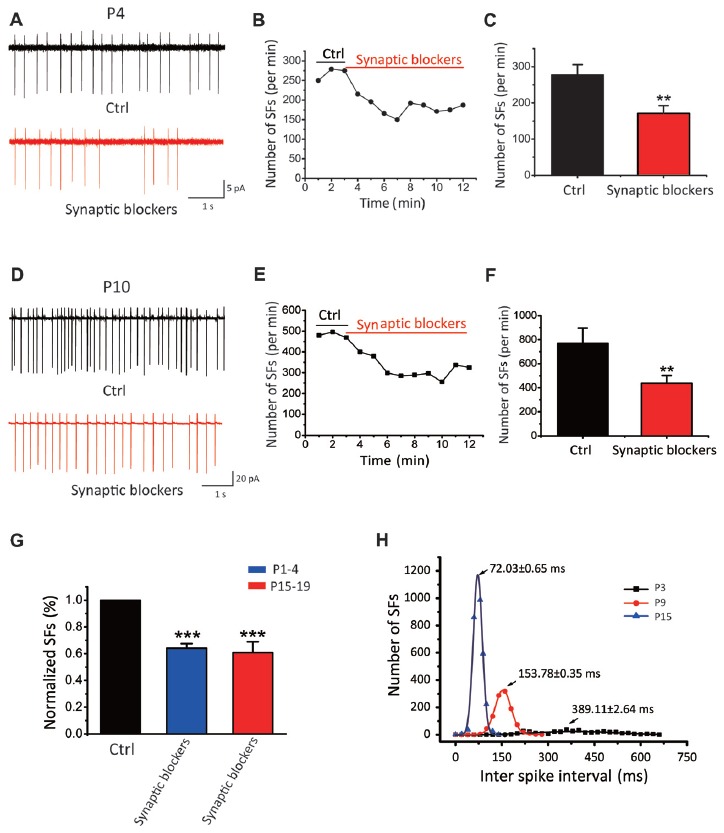
SFs are intrinsically driven and augmented by synaptic inputs in auditory central neurons. **(A–F)** Examples showing SFs before and after blocking synaptic inputs with a cocktail of NBQX, APV, bicu and stry in P4 **(A–C)** and P10 **(D–F)** auditory central neurons; Time courses of representative recordings from neurons shown in **(A,B)** are plotted in (**B,E)**. Comparisons of the average frequency of SFs in normal ACSF and synaptic blocker are shown in **(C,F)** for two age groups (P1–4 in control: 277.21 ± 28.69 spikes/min, synaptic blockers: 170.80 ± 28.69 spikes/min, P15–19 in control: 769.18 ± 126.48 spikes/min, synaptic blockers: 436.38 ± 65.46 spikes/min) recorded from 30 neurons in P15–19 mice. **(G)** Normalized firing rates in control and after application of synaptic blockers. **(H)** Distribution histograms of inter spike intervals for three typical IF(+) neurons at P3, P9 and P15, respectively. Smooth curves represent the best fits to the data with the Gaussian function in the form of: $\text{f}(\text{x})=\frac{1}{\sigma \sqrt{2\pi }}{\text{e}}^{\frac{-{\left(x-\mu \right)}^{2}}{2\sigma^{2}}}$, where μ denotes the x value at the center of the normal distribution, σ is the standard deviation from the x (P3: *μ* = 389.11 ± 2.64 ms, *σ* = 280 ± 50.96 ms; P9: *μ* = 153.78 ± 0.35 ms, *σ* = 51.82 ± 0.79 ms; P15: *μ* = 72.03 ± 0.65 ms, *σ* = 29.23 ± 1.92 ms).

Given all recorded neurons show sIPSCs and sEPSCs, we next investigated whether the spontaneous activity is triggered by synaptic inputs or generated intrinsically. We recorded from SF(+) CN neurons in cell-attached configuration before and after blocking excitatory glutamatergic and inhibitory GABAergic/Glycinergic inputs with a cocktail ACSF containing NBQX, APV, bicu and stry in slices from P1–P19 mice. As shown by two recordings from P4 and P10 neurons that exhibited very different initial discharge rates (268 vs. 483 spikes/min; Figures 2A,D), SFs were partially attenuated but not eliminated by these blockers (182 vs. 288 spike/min). Figures 2C,F summarize the mean frequencies of SFs before and after addition of synaptic blockers, being 277.21 ± 28.69 spikes/min and 170.80 ± 28.69 spikes/min for P1–4 neurons (*p* < 0.01, *n* = 11); and 769.18 ± 126.48 spikes/min and 436.38 ± 65.46 spikes/min for in P1–19 (*p* < 0.01, *n* = 30). Normalized data presented in Figure 2G suggested that more than 60% of SFs were driven intrinsically, while presynaptic inputs could activate glutamate and GABA receptors to enhance SFs in postsynaptic neurons. Figure 2H shows a histogram of inter-spike intervals for SFs of three active neurons from P3, P9 and P15, for which their distributions can be well described by the Gaussian function (see legend) with the mean inter spike interval decreasing from 389.5 ± 2.64 ms at P3 to 153.8 ± 0.73 ms and 72.04 ± 0.85 ms at P9 and P15, respectively. Standard deviation of their distributions decreases in parallel from 280 ± 50.96 ms at P3, to 51.82 ± 0.79 ms at P9 and 29.23 ± 1.92 ms at P15. These analyses indicate that SFs are rhythmic in nature and their rates tune up with development. The intrinsic firings (IFs) observed in the subset of P1–4 active neurons after blocking synaptic inputs (herein designated as IF(+) neurons vs. silent cells as IF(−) neurons) clearly demonstrate that early onset of active pacemaker conductances drives the rhythmic firing of postsynaptic neurons, but presynaptic inputs may work in concert to boost SFs during the sensitive period of development.

### Dominant Role of HCN Channels in Driving IFs of CN Neurons

**Figure 3:**
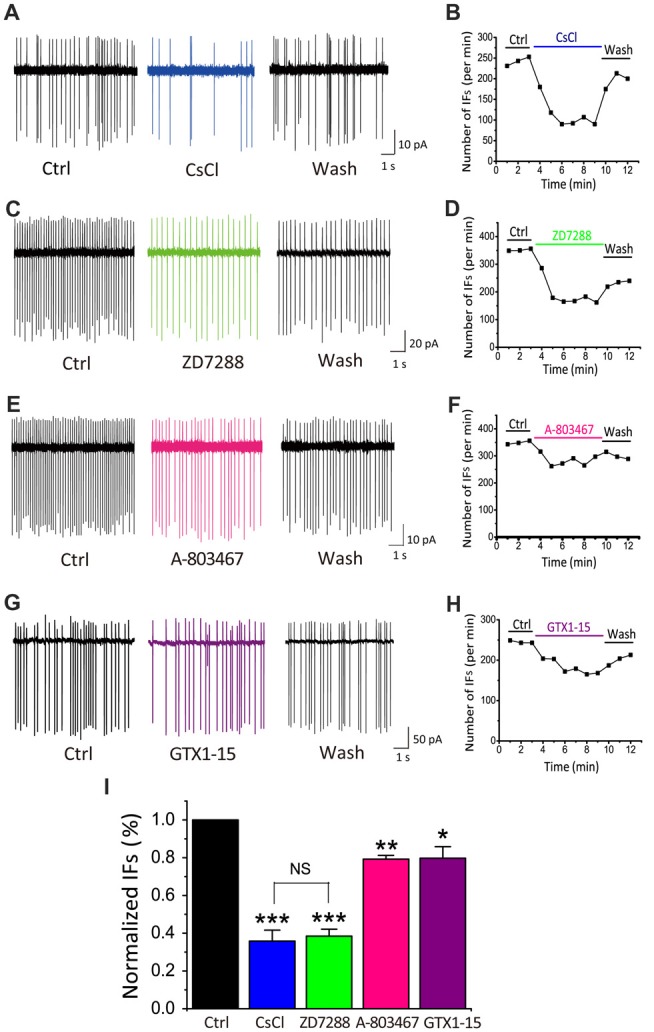
HCN channels play a dominant role in generating IFs over TTX-resistant sodium channels and T-type calcium channels in P5–10 CN neurons. **(A–H)** Example traces and time course are shown for control (in the presence of synaptic blockers), subsequent application and washout of HCN blockers CsCl (2 mM), ZD7288 (40 μM), or TTX-resistant sodium channel blocker A-803478 (10 μM) or T-type calcium channel blocker GTX1–15 (0.01 μM). The control solution was applied for 3 min, followed by the bath application of different blockers for 6 min and a washout for another 3 min. Data was collected after 2 min drug application to achieve equilibrium. **(I)** Summary plot of the inhibition of each antagonist, showing significantly larger effects of HCN channels blockers (CsCl and ZD7288), as compared with other two pacemaker channels. Normalized data were collected by taking the average of spikes for each 1-min bin during 6 min drug application.

Among the most common pacemaker conductances in central neurons, HCN channels have been identified in a number of sensory systems including the visual (Demontis et al., 2002), auditory (Cuttle et al., 2001; Shaikh and Finlayson, 2005) and vestibular systems (Almanza et al., 2012; Yoshimoto et al., 2015). Within the inner ear, HCN mediated I_h_ currents have been shown in vestibular (Chabbert et al., 2001) and auditory neurons (Mo and Davis, 1997; Yi et al., 2010). Unlike most other voltage-gated channels, the activation of HCN channels is controlled through dually interdependent membrane hyperpolarization and cyclic nucleotide (i.e., cAMP and cGMP) binding to their cytoplasmic domain (Kusch et al., 2010; Wu et al., 2011). To determine whether HCN channels contribute to the initiation of IFs, we applied two HCN pharmacological antagonists: CsCl (2 mM) and ZD7288 (40 μM; BoSmith et al., 1993; Harris and Constanti, 1995), and analyzed their effects on IFs in the presence of the same cocktail of synaptic blockers. Figures 3A,C show two examples of IF(+) neurons, in which the firing rate was 242.3 spikes/min and 351.6 spikes/min in control, but decreased to 148 spikes/min and 171.2 spikes/min after applications of CsCl and ZD7288, respectively. These effects were reversible upon washout as illustrated in Figures 3B,D.

TTX-resistant sodium channels and T-type calcium channels can also contribute to neuronal excitability and participate in the generation of pacemaker activities (Oka, 1995; Stevens et al., 2001; Felix et al., 2011; Kopp-Scheinpflug et al., 2011). We next investigated whether these channels were also involved in promoting IFs. Blockers of TTX-resistant sodium channels and T-types calcium channels, A-803467 and GTX1–15 were used in this experiment at concentrations described previously (Chauhan et al., 2017). As shown in Figure 3E, we found that after 6 min A-803467 (10 μM) application, the frequency of IFs decreased from 349 spikes/min to 275 spikes/min. Similarly, GTX1–15 (0.01 μM), a blocker for T type calcium channels, reduced the firing rate from 245 spikes/min to 181.8 spikes/min (Figure 3G). In both cases, the effects were largely reversible after washout (Figures 3F–H). To quantitatively compare the effectiveness of these blockers, we calculated the extent of changes in firing rates for each cell over the last 2-min bin in control and drug application and plotted the pooled data for relative reductions with respective blockers (Figure 3I). Statistical analyses revealed that HCN blockers produced significantly greater effects on the frequency of IFs (CsCl: 35.85 ± 5.77% of control, *p* < 0.001, *n* = 9; ZD7288: 38.45 ± 3.67% of control, *p* < 0.001, *n* = 10) than a TTX-resistant sodium channel blocker (A-803467: 79.24 ± 1.97% of control, *p* < 0.05, *n* = 5) or a T-type calcium channel blocker (GTX1–15: 79.74 ± 6.17%, *p* < 0.05, *n* = 7). Collectively, these results led us to conclude that HCN channels likely play a dominant role in generating IFs in central auditory neurons.

**Figure 4:**
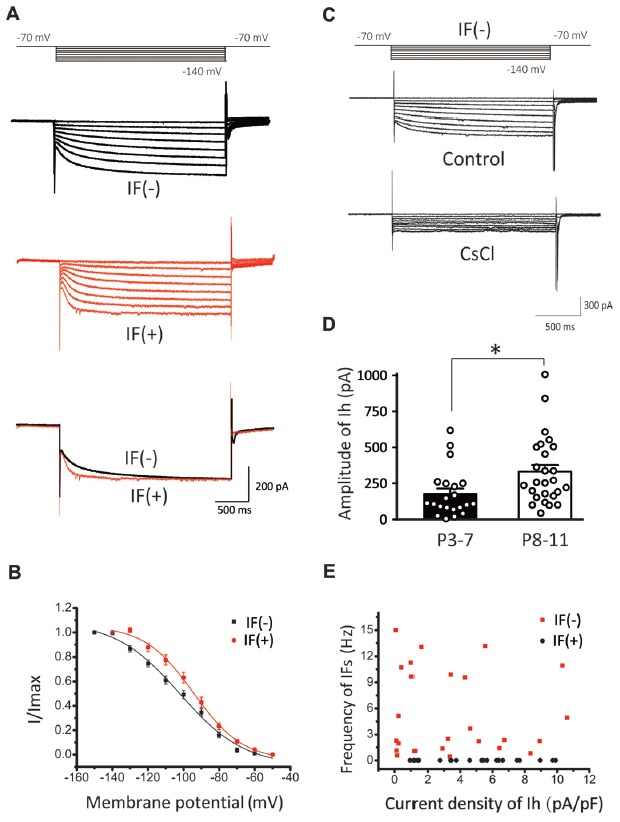
Comparison of I_h_ in IF(+) and IF(−) neurons. **(A)** Upper and middle panel: representative I_h_ recorded from IF(+) and IF(−) neurons. The membrane potential was held at −60 mV before different voltage steps ranging from −70 to −140 mV (in −10 mV increment) for 2 s as shown by the protocols above current traces. Lower panel: comparison of activation kinetics of HCN currents evoked at −140 mV from IF(+) and IF(−) neurons. **(B)** Activation curve of I_h_ in CN neurons from IF(+) and IF(−) neurons, fit with the Boltzmann function. **(C)** Representative I_h_ in IF(−) neuron, which can be blocked by CsCl. **(D)** The plot of I_h_ amplitude and age groups (P3–7 and P8–11). **(E)** Scattered plot of IFs frequency against I_h_ current density for both IF(+) and IF(−) neurons.

To further explore the relationship between HCN channels and IFs, we next recorded I_h_ in IF(+) and IF(−) neurons by a series of hyperpolarizing voltage steps (ranging from −70 mV to −140 mV in −10 mV increment). We found that both types of neurons exhibit I_h_ (Figure 4A), but their voltage-dependent activation and gating kinetics were evidently different. Superimposed traces of I_h_ evoked at −140 mV showed that IF(+) neurons utilize HCN channels with faster activation kinetics than those in IF(−) neurons (Figure 4B). To quantitatively describe these differences, we plotted normalized steady-state current-voltage relationships (I–V), and fit two sets of I-V curves with the Boltzmann function (*I/I*_max_ = 1/(1 + exp [(*V*_0.5_−*V*_m_)/*k*])), where *I* is the current, *I*_max_ is the maximal current, *V*_m_ is the membrane potential, *V*_0.5_ is the half-activation voltage, and *k* is the slope factor. As summarized in Figure 4B, activation curve was significantly right-shifted toward more depolarized potentials in IF(+) neurons, with V_0.5_ being −94.25 ± 1.27 mV, compared to −101.95 ± 2.24 mV in IF(−) neurons (*p* < 0.01, *n* = 11) while the slope factor was comparable (IF(+), *k* = 13.8 ± 1.42; IF(−), *k* = 18.21 ± 2.76; *p* > 0.05, *n* = 11). In both cases, these currents were sensitive to a low concentration of CsCl (2 mM), confirming that HCN channels mediate these current (Figure 4C). Amplitude of I_h_ was increased in two age groups (P3–7: 178.44 ± 36.37 pA; P8–11: 331.91 ± 48.22 pA, *p* < 0.05, *n* = 26), indicating an age-dependent up regulation of HCN channel density (Figure 4D). Interestingly, the current density of I_h_ appeared to have no strong correlation with the frequency of IFs on the hyperpolarization-activated currents in IF(−) neurons (Figure 4E). These results indicated that HCN channels in CN neurons are heterogeneous, with fast activating I_h_ specifically associated with IF(+) neurons whereas slow activating I_h_ are prominently present in IF(−) neurons but less effective in pacemaking.

### IFs Are Regulated by Exogenous and Endogenous Cyclic Nucleotide

**Figure 5:**
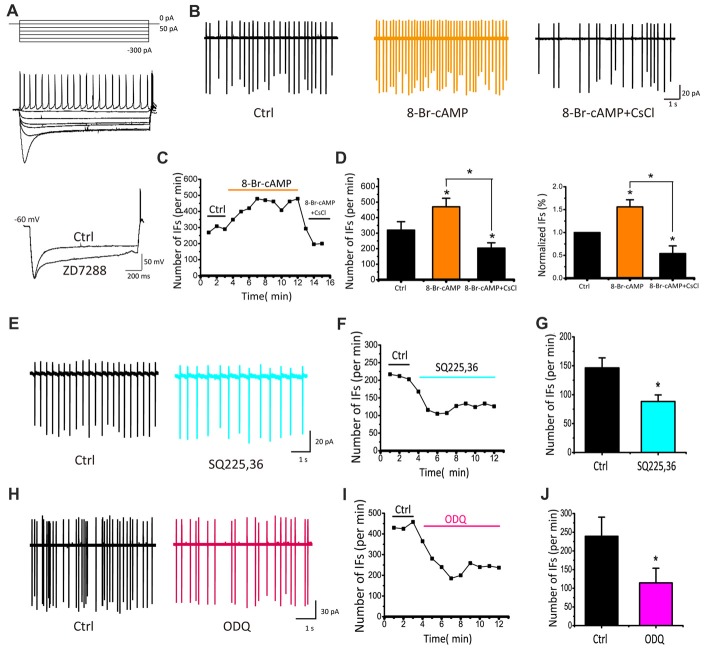
IFs are regulated by cyclic nucleotide in P5–10 CN neurons. **(A)** Voltage responses produced with 2 s hyperpolarizing pulses from −300 pA to 0 pA in 50 pA increments showing a depolarizing sag and rebound action potential, both of which are attenuated by ZD7288. **(B)** Example traces and time course of recording IFs before and after 8-Br-cAMP and CsCl applications. Spike number per minute recording from **(B)** is plotted against time in **(C)**. **(D)** Absolute and normalized rates of IFs for five cells are plotted for 8-Br-cAMP and 8-Br-cAMP+CsCl, respectively. Vertical bars indicate ±SE *p < 0.05. The control solution was applied for 3 min, followed by the bath application of 8-Br-cAMP for 6 min and CsCl application for another 3 min. Data was collected after 2 min drug application to achieve desired concentration. **(E–H)** Typical recordings of IFs before and during application of inhibitor for the adenylyl cyclase (AC) or guanylyl cyclase (GC), SQ225, 36 (50 μM) or ODQ (30 μM), showing that lowering endogenous level of cyclic nucleotides attenuates IFs. **(F–I)** Time course of representative recordings from neurons shown in **(E,H)**. The mean rates of IFs for five cells are plotted for SQ225, 36 and ODQ, respectively in **(G–J)**. Normalized data were collected by taking the average of spikes for each 1 min bin during 6 min drug application.

To directly demonstrate physiological roles and modulation of I_h_, we performed whole-cell current-clamp recordings by injecting a series of current steps into CN neurons (ranging from −300 pA to +50 pA in +50 pA increments) for 2 s. When the recorded neuron was hyperpolarized, the membrane potential displayed a “sag” and then depolarized towards the resting level and rebounded upon termination of the hyperpolarization current step, characteristic of I_h_. Application of ZD7288 (40 μM) significantly blocked both the sag and rebound, confirming HCN channels play direct roles in regulating intrinsic excitability of developing CN neurons (Figure 5A). To further examine the effects of modulating HCN channels in CN neurons, we recorded IFs in cell-attached mode and tested the effect of 8-Br-cAMP, a cell-permeable cAMP analog, known to potentiate I_h_ currents by directly binding to the intracellular cyclic nucleotide motif of HCN channels. At the concentration of 0.1 mM, application of 8-Br-cAMP for 9 min increased the rate of IFs from 289.4 spikes/min to 436.4 spikes/min (Figure 5B). Subsequently, we added CsCl to the perfusion solution and found that it significantly reduced the rate of IFs to a level below the control (229.7 spikes/min), implying that endogenous cyclic nucleotides must be high enough to tonically activate I_h_. Figure 5C shows the time-dependent change in spike frequency from an IF(+) neuron. Both the absolute and normalized changes were pooled and presented in Figures 5D, in which the rate of IFs after 8-Br-cAMP application was significantly elevated by more than 50% (control: 319.67 ± 54.06 spikes/min; 8-Br-cAMP: 469.53 ± 55.28 spikes/min or 155.98 ± 15.58% of control, *p* < 0.05, *n* = 5), whereas subsequent application of CsCl decreased it to 203.78 ± 34.20 spikes/min, much lower than the basal rate (or 53.93 ± 16.75% of control, *p* < 0.05, *n* = 5). These results demonstrated that exogenous cyclic nucleotide such as cAMP can induce increase in IFs via the enhancement of HCN channels.

Adenylyl cyclase (AC) and guanylyl cyclase (GC) are widely expressed enzymes in prokaryotes and eukaryotes that catalyze the production of endogenous cAMP and cGMP from adenosine and guanosine triphosphates. To manipulate the activity of HCN channels by endogenous pathways, we used inhibitors of AC and GC to regulate the level of cAMP or cGMP, and then examined their effects on IFs. We found that both SQ22536 (50 μM) and ODQ (30 μM), potent inhibitors for AC and GC respectively, decreased the rate of IFs significantly as shown in two examples of recordings from IF(+) neurons in Figures 5E,H. On average, the frequency of IFs was decreased from 146.47 ± 17.30 to 88.30 ± 11.17 spikes/min by SQ22536 (*p* < 0.05, *n* = 5; Figure 5G), and from 239.17 ± 51.14 to 114.42 ± 39.21 spikes/min by ODQ (*p* < 0.05, *n* = 4; Figure 5J). These convergent results imply that I_h_ mediated by HCN channels is likely involved in regulating IFs independently from synaptic inputs, but is influenced by endogenous levels of cAMP/cGMP.

### HCN1 Knockout Attenuates the Amplitude of Native I_h_ and IFs in CN Neurons

**Figure 6:**
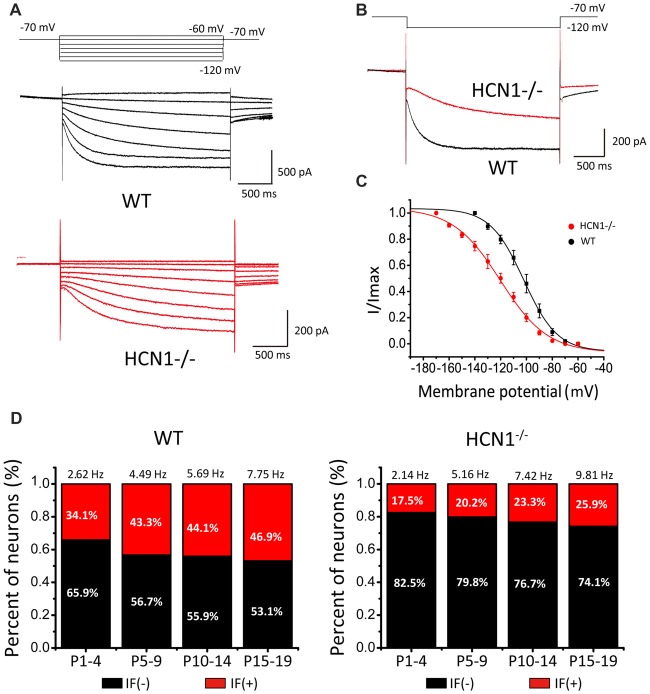
Hyperpolarization- and cyclic nucleotide-gated channel 1 (HCN1) knockout attenuates the amplitude of native I_h_ and IFs in CN neurons. **(A)** Representative I_h_ recorded from wild-type (WT) and HCN1 deficient mice. The membrane potential was held at −70 mV before different voltage steps ranging from −60 mV to −120 mV (in −10 mV increment) for 2 s as shown by the protocols above current traces. **(B)** Comparison of HCN currents evoked at −120 mV from CN neurons from WT and HCN1^−/−^ mice showing smaller and slower I_h_ in HCN1^−/−^ neurons. **(C)** Activation curve of I_h_ in CN neurons from WT and HCN1^−/−^ mice, fit with the Boltzmann function. **(D)** Histograms that showing percentages of IF (+) neurons in different age groups. Numbers above bars are the frequencies of IFs for each age group.

HCN channels consist of four isoforms, HCN1–4 (Ludwig et al., 1998; Robinson and Siegelbaum, 2003), among which HCN1 is the most widely expressed and is particularly abundant in CN neurons. To test if HCN1 is responsible for native I_h_ currents as described, we performed whole-cell voltage-clamp recordings from CN neurons in HCN1 knockout mice (HCN1^−/−^) and wild-type (WT) littermates. Representative I_h_ currents recorded in CN evoked by a series of hyperpolarizing voltage steps are depicted in Figure 6A. We found both current amplitude and activation kinetics of I_h_ were noticeably different in HCN1^−/−^compared with WT, being smaller in size at the same test potentials (steady-state amplitude of I_h_ at −120 mV; WT: 762.18 ± 78.12 pA; HCN1^−/−^: 399.12 ± 50.17 pA; Figure 6B). We found that the activation curve was shifted toward more hyperpolarized potentials in HCN1^−/−^CN neurons (V_0.5_: WT, −102.06 ± 1.18 mV; HCN1^−/−^, −120.25 ± 1.70 mV, *p* < 0.05, *n* = 5; *k*: WT, 13.02 ± 1.30; HCN1^−/−^, 18.36 ± 1.95, *p* > 0.05, *n* = 6; Figure 6C). These differences between WT and HCN1^−/−^neurons indicated HCN1 channels are the main mediator of native I_h_ channels, albeit other HCN channels may also contribute to I_h_, or compensate for the loss of HCN1, in line with at least two populations of HCN channels associated with IF(+) and IF(−) neurons in WT CN neurons as depicted in Figure 4.

Because of the significant left-shift of the activation curve, one can predict that deletion of HCN1 would attenuate IFs. To test this, we made a large number of recordings to quantify the fraction of IF(+) or IF(−) CN neurons and their firing rates in WT and HCN1^−/−^ mouse littermates at various developmental stages. Results from a total of 421 neurons showed a much smaller proportion of IF(+) neurons from HCN1^−/−^ mice than those from WT mice for all four age groups (i.e., P1–4, P5–10, P11–14 and P15–19). Data from both HCN1^−/−^ and WT mice were sorted and summarized in Figure 6D, showing that the fraction of neurons with IFs increases with age in both genotypes but diverges in their relative weight. The fraction of IF(+) neurons in WT mice was 34%, 43.2%, 44.1% and 53.1% (IF(+): *n* = 17, 16, 15, 8; IF(−): *n* = 33, 21, 19, 12 in four age groups), significantly higher than that in HCN1^−/−^mice, being 17.5%, 20.3%, 23.3% and 25.9% (IF(+): *n* = 14, 18, 14, 14; IF(−): *n* = 66, 71, 46, 40) for four age groups, respectively (P1–4: χ^2^ = 7.2, *p* < 0.01; P5–10: χ^2^ = 7.612, *p* < 0.01; P11–14: χ^2^ = 4.348, *p* < 0.05; P15–19: χ^2^ = 5.694, *p* < 0.05). Pairwise comparisons in different age groups indicated the mean firing rates were comparable for the same age groups. Firing rates between WT and HCN1^−/−^ mice were not significant among four age groups (P1–4: WT: 2.62 Hz, KO: 2.14 Hz, *p* > 0.05, *n* = 17; P5–9: WT: 4.49 Hz, KO: 5.16 Hz, *p* > 0.05, *n* = 16; P11–14: WT: 5.69 Hz, KO: 7.42 Hz, *p* > 0.05, *n* = 15; P15–19: WT: 7.75 Hz, KO: 9.81 Hz, *p* > 0.05, *n* = 12). In IF(+) CN neurons from HCN1^−/−^ mice, addition of CsCl or ZD7288 will further dampen down and in some cases completely eliminate IFs, suggesting other HCNs can partially compensate for the loss of HCN1. We found five out of six IF(+) neurons from P1–4 HCN1^−/−^ mice were silent after perfusion of ZD7288. The remaining neuron exhibited 45% reduction in its firings by ZD7288, which were completely eliminated by application of TTX-resistant sodium channel blocker (A-803467). These observations indicate that HCN1 is a dominant but not exclusive pacemaker, and other pacemakers (e.g., HCN2–4, TTX-resistant sodium channel) could partially compensate for the loss of HCN1 channels. These results provide the compelling evidence to demonstrate that HCN1 is a major role in driving IFs. Notably, the fraction of IF(+) neurons is increased during development in WT, but this developmental change is reduced in HCN(−/−), implicating the growing contribution of HCN1 channels in the generation of IFs as the auditory brainstem develops towards maturity.

### SFs Are Correlated With Accelerated Maturation of Membrane Excitability and Synaptic Function

**Figure 7:**
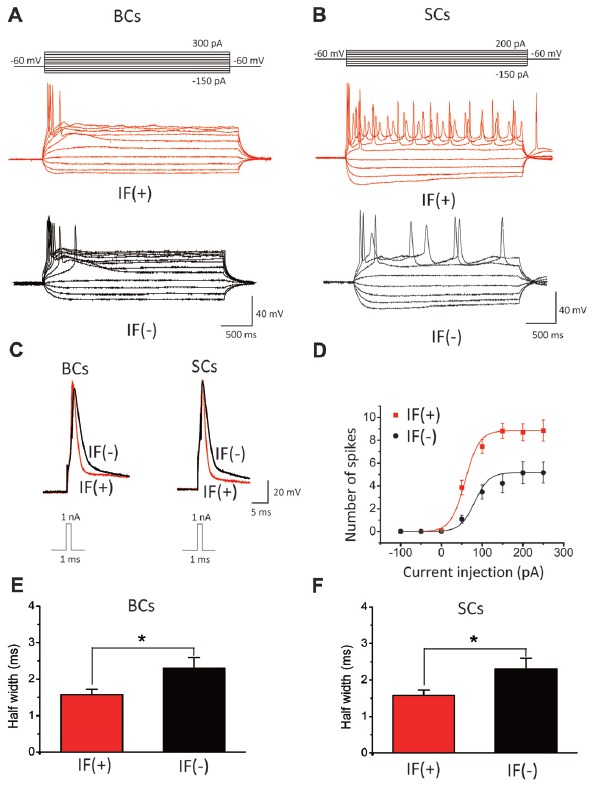
Neurons with IFs exhibit higher level of membrane excitability in P4–7 CN neurons. **(A,B)** Example traces of action potentials (APs) evoked by depolarizing currents recorded from IF(+) and IF(−) bushy cells (BCs) and stellate cells (SCs). **(C)** Half-width of action potentials evoked by 1 nA current injection in 1 ms were compared in IF(+) and IF(−) neurons, showing much narrower waveform of APs in IF(+) neurons (BCs: IF(+): 1.29 ± 0.14 ms, *n* = 7; IF(−): 2.07 ± 0.15 ms, *n* = 9, *p* < 0.05; SCs: IF(+): 1.58 ± 0.14 ms, *n* = 27; IF(−): 2.3 ± 0.29 ms, *n* = 14, *p* < 0.05). The input resistances for IF(+) and IF(−) cells (measured at −60 mV with a 5 mV hyperpolarization) are comparable for both types of neurons (SCs: IF(+), 153.5 ± 21.24 MΩ, *n* = 9 vs. IF(−), 148.95 ± 21.69 MΩ, *n* = 12, *P* > 0.05; BCs: IF(+) 191.35 ± 10.46 MΩ, *n* = 6; IF(−) 193.75 ± 25.42 MΩ, *n* = 8); **(D)** The number of spikes generated by various depolarization steps in IF(+) and IF(−) stellate cells. Data were fit with the Boltzmann function. **(E,F)** Summary plot of the half-width of APs in bushy and stellate cells, showing neurons with IFs have briefer APs.

To further study the functional relevance of spontaneous activity, we compared the excitability of neonatal IF(+) and IF(−) neurons in WT mice. On the basis of their distinct firing patterns in response to sustained depolarization, we broadly classified two groups of neurons, those that respond with a single spike only and those with a multiple spike response to a single current step. These single-spike and multi-spike firing patterns are well-established in mature CN neurons, resembling those of bushy cells and stellate cells at maturity (BCs and SCs), respectively (Oertel et al., 1990; Wang et al., 2010; Song et al., 2012). In slices from P4–P7 mice, we examined input-output relationships by first injecting a small hyperpolarization current (<50 pA) to arrest firings of IF(+) neurons and manually setting the same membrane potential (i.e., −60 mV) for both IF(+) and IF(−) neurons from their resting potentials (i.e., −50 to −55 mV) before delivering a series of current steps (−150 to +300 pA, 50 pA increments; Figure 7A,B). Consistent with previous studies, BCs typically fire single spike near the onset of depolarizing current steps while SCs increases the number of spikes as a function of depolarizing current magnitude. In both cases, IF(+) neurons appeared to have a different spike waveform from IF(−) neurons. To directly compare the spike waveform in IF(+) and IF(−) neurons, we injected a brief pulse of current (1 nA current in1 ms from the set membrane potential of −60 mV) to evoke single spikes. We found that the waveforms of both IF(+) BCs and SCs were much narrower than those of IF(−) neurons (Figures 7C,E, BCs: IF(+): 1.29 ± 0.14 ms, *n* = 7; IF(−): 2.07 ± 0.15 ms, *n* = 9, *p* < 0.05; Figures 7C,F, SCs: IF(+): 1.58 ± 0.14 ms, *n* = 27;I F(−): 2.3 ± 0.29 ms, *n* = 14, *p* < 0.05). Given that the half-width of spikes in P16–21 BCs and SCs reported in the literature is in the sub-millisecond range (Zhong et al., 2014), our results suggested that IF(+) neurons show more accelerated progress in their development towards mature spike waveform and firing phenotypes than quiescent neurons. This is further supported by comparing the input-output relationships of IF(+) and IF(−) SCs (Figures 7B,D). When the number of spikes in response to each current step was counted and plotted, we found that IF(+) SCs showed higher number of spikes in response to the same magnitude of current injections, as quantitatively described by fitting to input-output curves with the Boltzman function (i.e., *S/S*_max_ = 1/(1 + exp[(I_0.5_−I)/k])) where *S* is the spike number, *S*_max_ is the maximal spike number, *I* is the current, *I*_0.5_ is the half maximal current magnitude to evoke *Smax*, and *k* is the slope factor. (*Smax*: IF(+): 8.85 ± 0.93, IF(−): 5.17 ± 0.91, *p* < 0.05, *n* = 4; *I*_0.5_: IF(+): 56.36 ± 3.02 pA, IF(−): 82.72 ± 6.33 pA, *p* < 0.05; *k*: IF(+): 21.73 ± 2.96 pA, IF(−): 28.58 ± 5.60 pA, *p* > 0.05, *n* = 4) whereas input resistances for IF(+) and IF(−) are comparable (see Figure 7 legend)). These results suggest that the IF(+) neuron subset, in comparison to IF(−) neurons in the same slices from P4–7 mice, is associated with more rapid up regulation of their intrinsic membrane excitability, accelerating developmental remodeling of spike waveforms and firing phenotypes.

**Figure 8:**
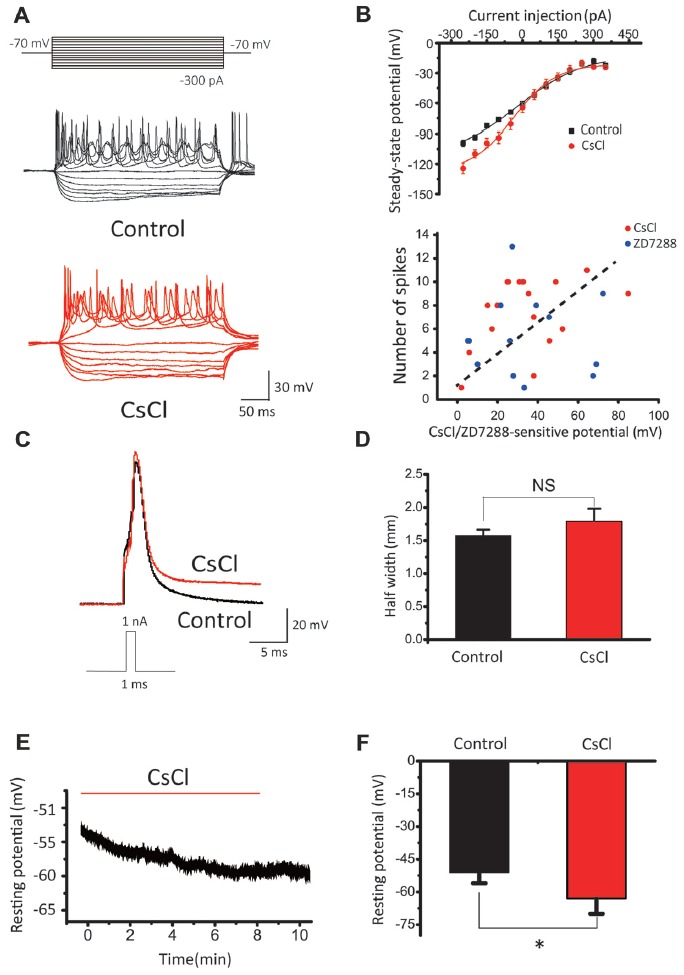
The effects of HCN blockers in membrane properties. **(A)** Representative recordings in response to current step injections in control and CsCl. **(B)** Upper Panel: summaries of steady-state potentials measured within the last 5 ms range of the evoked potentials in control and CsCl. Lower Panel: relationship between CsCl/ZD7288 sensitive component of membrane potential at −140 mV and maximal spike number among the same population of cells. **(C)** Superimposed APs evoked by 1 nA, 1 ms current pulses in control (black) and CsCl (red). **(D)** Summary plots of the effect of CsCl on the half-width of evoked APs. **(E)** Example of changes in resting membrane potential following application of CsCl. **(F)** Summary plots of the effect of CsCl on RMP.

To investigate the roles of I_h_ in passive and active membrane properties, we further compared spike number, steady-state potential, half-width of evoked action potential and resting membrane potential before and after addition of HCN blockers in SCs. Notably, CsCl significantly reduced spike number evoked by current step injections (Figure 8A), and increased the slope factor of steady-state membrane potentials (within the last 5 ms of each step) from 89.81 ± 14.24 to 139.99 ± 28.62 (Figure 8B, *upper panel*). In addition, when CsCl/ZD7288-sensitive component of membrane potential (“sag”) as subtracted before and after addition of CsCl or ZD7288 is plotted against the maximal spike number from the same cells (Figure 8B, *lower panel*), there appears to be a trend of positive correlation between them, albeit not statistically significant. I_h_ blockers had marginal effects on the waveform of single action potentials (APs) evoked by brief current steps from a set potential of −60 mV, with their half-width being changed by CsCl from 1.57 ± 0.09 ms to 1.79 ± 0.19 ms, *n* = 11, *p* > 0.05, Figures 8C,D). In contrast, the resting membrane potentials were significantly hyperpolarized from −51 ± 5 mV to −63 ± 7 mV (*n* = 11, *p* < 0.05, Figures 8E,F). These results indicate that HCN channels play a major role in defining the resting membrane potential and intrinsic excitability of CN neurons to differentially drive the phenotypes of IF(+) and IF(−) neurons during their developmental maturation.

**Figure 9:**
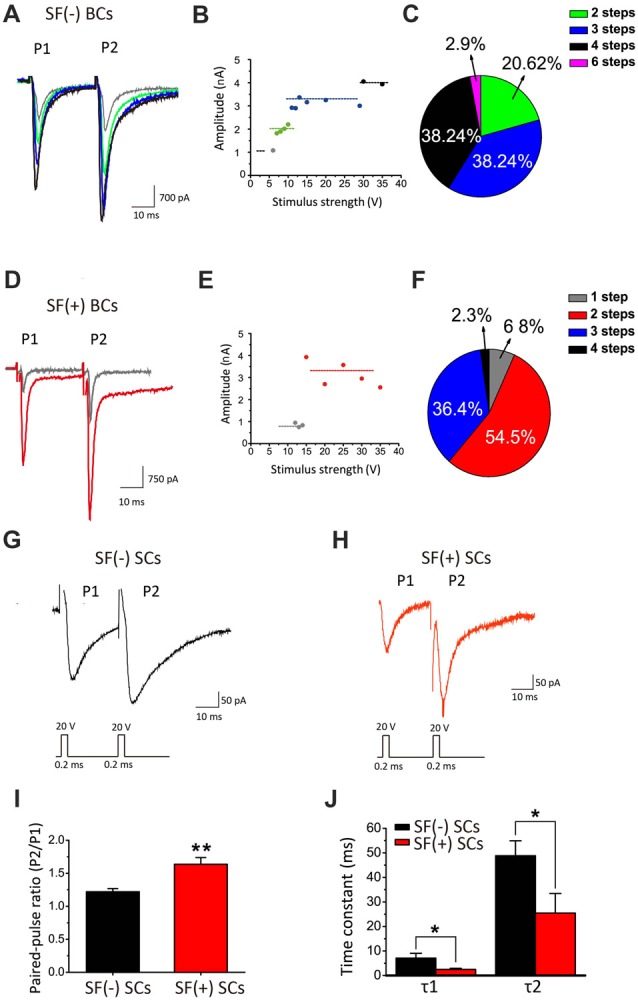
SFs are correlated with accelerated maturation of functional connectivity and remodeling. **(A)** Example traces of evoked EPSCs recorded from a SF(−) BC. **(B)** The amplitude of eEPSCs in different stimulus strength for this neuron increased in four distinct steps as shown in **(A)**. **(C)** Pie chart illustrating the relative fraction of BCs with different number of stepwise changes in response to increasing stimulation intensity. **(D)** Example traces of evoked EPSCs recorded from a SF(+) BC. **(E)** The amplitude of eEPSCs in different stimulus strength in this BC showed two stepwise increases. **(F)** Pie chart showing significant increases in the fraction of bushy cells with less than three stepwise changes when these neurons are primed by SFs. **(G,H)** Example traces of eEPSCs recorded from SF(+) and SF(−) SCs. **(I)** Mean value of paired-pulse ratio (PPR) of EPSC charge in SF(+) and SF(−) SCs. **(J)** Average time constants (τ1 and τ2) from exponential fits to the decay phase of EPSCs from SF(+) and SF(−) SCs. All recordings were made in the presence of stry and bicu.

It has been well established that synaptic connections also undergo significant remodeling, including pruning, during development. Mature BCs are typically innervated by fewer axosomatic nerve terminals than immature ones (i.e., the end bulb of Held; Ryugo et al., 2006; Cao and Oertel, 2010). This raises the possibility that multiple auditory nerve endings may be present on the soma during early development, but some of these will eventually dominate while other synaptic connections are eliminated. To determine whether synaptic pruning takes place and is affected by SFs, we performed whole-cell voltage-clamp recordings of evoked EPSCs from BCs by stimulating the auditory nerve stub with a bipolar electrode (i.e., paired-pulse paradigm) after blocking inhibitory inputs in WT mice. Typically, when the stimulation intensity gradually increase, we found that EPSCs were elicited in an all-or-none manner, but their amplitude increased in a stepwise manner (Figures 9B,E), consistent with the idea that each stepwise increment reflects the recruitment of additional afferent fiber with different activation thresholds. Contrasting two examples of recordings from the same slice (Figures 9A,D), we noted that the SF(−) BCs had at least four inputs where only two synaptic inputs were detected in SF(+) BCs, indicating BCs with SFs may have more advanced synaptic pruning than those without. Among 34 BCs, 61.3% of SF(+) neurons showed only 1–2 stepwise increments while in contrast, 76.5% of SF(−) neurons had 3–4 steps (Figures 9C,F). These results indicated that peripheral inputs undergo developmental remodeling in connectivity, and that SFs in postsynaptic neurons may be engaged in presynaptic pruning to refine the synaptic connectivity.

SCs receive polysynaptic excitatory inputs on their dendrites from both auditory nerves and other local excitatory inputs within CN, making it difficult to study pruning of presynaptic inputs onto these cells. Instead, we examined synaptic properties between SF(+) and SF(−) SCs by comparing paired-pulse ratio (PPR) of evoked EPSCs, which can serve as an indirect readout of the release probability (Pr). We found that SF(+) SCs showed significantly higher PPR (PPR: 1.64 ± 0.10, *n* = 12) than SF(−) SCs (PPR: 1.22 ± 0.55; *n* = 13, *p* < 0.05), implying SF(+) SCs have lower initial Pr at their presynaptic terminals (likely due to spike narrowing) as synapses mature. Notably, the time course of EPSCs was also much faster in SCs with SFs than those without, when the decay phase of the first EPSCs were fit with the double-exponential function (SF(+) VS SF(−): τ_1_: 2.645 ± 0.52 ms vs. 6.09 ± 1.84 ms, *n* = 13, *p* < 0.05; τ_2_: 28.46 ± 7.89 ms vs. 41.77 ± 7.18, *n* = 13, *p* < 0.05; Figures 9I,J), suggesting postsynaptic glutamate receptors may have also undergone developmental subunit switch to accelerate gating. Taken together, our results from principal CN neurons indicated that SFs in postsynaptic neurons are correlated with accelerated remodeling of presynaptic connectivity and function, and postsynaptic membrane excitability, potentially promoting the maturation of peripheral inputs to the first-order neurons in central nuclei.

## Discussion

This study demonstrates that a subset of the first central neurons in the auditory brainstem is capable of firing spontaneously, independent of synaptic inputs, and that this spontaneous activity during the early postnatal period is intrinsically driven by HCN channel-mediated I_h_. We further demonstrated that neurons with SFs exhibit accelerated development in their intrinsic excitability, spike waveform, and pruning of peripheral inputs as well as synaptic functionality. These results suggest that SFs in CN neurons may potentially engage in the cross-talk between neurons in upstream and downstream nuclei in order to establish proper wiring and refinement of the necessary circuitry of the auditory brainstem before the onset of hearing. Our findings complement previous findings that spontaneous activities in peripheral sensory neurons like IHCs are instructive (Tritsch et al., 2007). However, we propose that IFs in first-order central neurons driven by postsynaptic pacemaker conductances such as I_h_ may be critical for echoing presynaptic inputs, potentially promoting the formation and remodeling of elaborate neural circuits and networks in the developing brain.

### Spontaneous and Intrinsic Activity in Central Auditory Neurons

In early development, the nervous system produces patterned SFs. Many lines of evidence implicate SFs in driving synaptic plasticity and circuit maturation (Kerschensteiner, 2014). In the auditory system before the onset of hearing, there is strong evidence for the existence of bursts of action potentials in IHCs, SGNs, CN and MNTB. These events are triggered and synchronized by local ATP release (Tritsch et al., 2007), although spike generation in IHCs may be intrinsic to the cell itself (Johnson et al., 2011). The prevailing view is that these spontaneous activities in IHCs and SGNs, before the opening of ear canals, play instructive roles in establishing the connectivity of central auditory neurons, and remodeling of circuits and networks for sound processing during the early development. In this study, we discovered that postnatal CN neurons exhibit SFs as early as P0, and a majority of these neurons demonstrate a rhythmic spike firing pattern rather than bursts of activity (Figure 1), supporting the existence of intrinsic pacemaker conductances that drive regular spiking. Indeed, after pharmacologically blocking all synaptic inputs, we found that this subset of neurons remained active, and exhibited the firing characteristics of intrinsic pacemakers (Figure 2). As described for many brain areas, such as the spinal cord and sub thalamic nucleus (Mao et al., 2001; Streit et al., 2001; Atherton et al., 2008), neonatal pacemaker neurons are thought to be instrumental to synchronizing network activity and the subsequent establishment of synaptic connectivity (Li and Baccei, 2011). Intrinsically active pacemaker neurons arising from pluripotent stem cells were also previously shown to be a key element in regulating oscillatory activities in neuronal assemblies (Illes et al., 2014). Based on these findings, we suggest that the first-order central neurons with IFs are potentially the most excitable targets for initial peripheral innervation.

### HCN Channels Play a Critical Role in the Generation of IFs in Auditory Neurons

HCN channels mediate nonselective cation inward currents and are activated during membrane hyperpolarization, exhibiting low activation kinetics that are modulated by intracellular cAMP (Pape, 1996; Accili et al., 2002). These channels actively contribute to various physiological properties and functions of neurons, including neuronal pacemaker activity, setting of resting membrane potential and dendritic integration of synaptic inputs (Bender et al., 2007; Kim and Holt, 2013; Thuault et al., 2013; Chen et al., 2015). Auditory brainstem neurons express HCN channels more abundantly than most other neuron populations (Bal and Oertel, 2000; Cuttle et al., 2001; Leao et al., 2006). In the auditory nervous system, HCN currents have been characterized in cells of various tissue types, such as vestibular hair cells, inner hearing cells, and ventral and dorsal CN neurons (Cao and Oertel, 2011; Rusznák et al., 2013). In this study, we showed the existence of HCN channels in CN neurons by evaluating their known biophysical properties (Figures 6A–C). Several studies have shown an increase in current density of HCN currents across the developmental period (Biel et al., 2009; Almanza et al., 2012; He et al., 2014), in line with our observations of increasing IFs with age (Figure 6D). Application of exogenous 8-Br-cAMP, a membrane permeable analog of cAMP, significantly increased the frequency of IFs (Figure 5), while pharmacologically blocking HCN channels by CsCl and ZD7288 did the opposite (Figure 3). More importantly, pharmacological inhibition of AC and GC could attenuate the frequency of IFs, suggesting HCN channels are tonically activated by high endogenous cAMP/cGMP levels to regulate pace making and the resting membrane potential (Figure 3). These channels are activated in the hyperpolarization stage of the preceding action potential. The inward current mediated by HCN channels leads to the rebound of the membrane potential closer to the activation threshold for Na^+^ channels, making it a robust trigger for regenerating the next action potential (Hassfurth et al., 2009; Almanza et al., 2012). Our results showing that blockers of HCN channels (CsCl and ZD7288) significantly decreased the frequency of IFs reinforces the notion that HCN channels play a dominant role over the other pacemaker channels in contributing to the generation of IFs in auditory neurons. Indeed, when the application sequence of blockers for I_h_, TTX-resistant Na^+^ channel and T-type VGCCs was reversed, we found CsCl attenuated IFs more effectively than other blockers. Although these pharmacological blockers have limited specificity at the concentrations used for slice preparation, the finding that CN neurons from HCN1 knockout mice exhibit much lower fraction of IF(+) neurons compared to those from WT mice builds a compelling argument for the dominant role of I_h_ over other pace making conductances in triggering IFs in the early development stage. Given smaller current amplitude and slower activation kinetics of I_h_ in HCN1−/− mice, we postulate that unknown developmental compensations by other channels may underlie the slight up regulation of firing frequency in the subset of remaining IF(+)s in the knockout. However, our observations from WT mice showed that IF(+) neurons employ HCN channels with more positive voltage-dependence in activation and faster gating kinetics (i.e., reminiscent of HCN1 channels) than IF(−) neurons, implicating HCN1 channels as the most critical pacemaker. However, lack of strong correlation between HCN current density alone and the frequency of IFs (Figure 4E), and between the CsCl/ZD7288 sensitive component and maximal spike number in the same population of neurons (Figure 8B) suggests HCN channels are necessary but insufficient to determine the frequency of IFs, which is likely driven by synergistic actions of other voltage-gated conductances. Given the heterogeneity of cell types in CN, it is difficult to define if I_h_ is specifically enriched in certain types of neurons without reliable genetic and biophysical markers during early development (P1–4). However, our finding showing two populations (i.e., active and silent) of positively identified BCs or SCs at a later stage (P4–7) implies that up regulation of the fraction of spontaneously active neurons and their firing rates are not necessarily a cell-type specific phenomenon.

### Molecular Correlates of Native HCN Channels in Auditory Neurons

I_h_ affects membrane properties and neuron excitability in most auditory neurons (Bal and Oertel, 2000; Cuttle et al., 2001). However, the effect of I_h_ on trigging SFs and IFs in first-order auditory neurons has not yet been described. HCN isoforms (HCN1 toHCN4) of the I_h_ family of channels are differentially expressed in auditory neurons from cochlear nuclei to cortex (Monteggia et al., 2000), and most auditory neurons in the brainstem likely express all isoforms, especially HCN1, 2 and 4 (Koch et al., 2004; Kim and Holt, 2013). HCN1 channels have the fastest activation kinetics and most depolarized half-activation potential among the isoforms, whereas homomeric HCN4 channels show the slowest activation kinetics (Santoro et al., 2000). Homomeric HCN2 channels exhibit intermediate activation kinetics relative to HCN1 and HCN4. Previous work demonstrated robust expression of HCN1 and their functional currents in the CN (Cao and Oertel, 2011; Rusznák et al., 2013), but a direct link between I_h_ and spontaneous spiking has never been shown. We found that genetic deletion of HCN1 significantly slowed the activation kinetics of I_h_ and reduced the fraction of neonatal CN neurons with SFs, suggesting HCN1 is a major molecular component of native HCN channels. This is supported by our results in Figure 4B showing that the V_0.5_ was more depolarized in IF(+) neurons compared with IF(−) neurons, therefore we infer that HCN1 is probably a major subunit with faster activation kinetics to drive IFs. Although a small fraction of CN neurons from HCN1^−/−^mice still exhibited IFs, we found ZD7288 remained more effective in attenuating or eliminating IFs than blockers for TTX-resistant Na^+^ channels and T type VGCCs (data not shown). These results indicated that other HCN channels (e.g., HCN2 and HCN4) might partially compensate for the loss of HCN1, but their slower activation kinetics and more negative activation threshold preclude them from being as effective for pace making as HCN1. The inability for HCN2/4 and other pace making channels to fully compensate likely underlies the age-dependent divergence in the fraction of IF CN neurons between WT and HCN1 knockout mice (Figure 6D). These results indicate that HCN1 channels mediate a majority of native I_h_ in active neurons of the first central auditory nucleus and are crucial for the generation of IFs, synergistically boosting SFs along with ongoing synaptic inputs.

### The Potential Role of SFs in Remodeling of Neuronal Firing and Wiring

During the early developmental stage, neurons appear to be genetically programmed to specify their distinct phenotypes and migrate to stereotyped positions to establish an initial set of connections. Correlated neuronal activity between presynaptic inputs and postsynaptic neurons is thought to be the core mechanism enabling the refinement of morphological and functional connectivity: strong synapses are reinforced while weak ones are pruned (Marrs and Spirou, 2012; Yu and Goodrich, 2014). Our results showed that the first-order auditory neurons are spontaneously active, I_h_ being the intrinsic pacemaker, and that neurons with IFs display elevated excitability and accelerated pruning compared to neurons without IFs (Figure 9). However, it is puzzling that *in vivo* recordings from central auditory nuclei show burst activity patterns that strongly correlate with ATP-dependent calcium spikes in IHCs (Tritsch et al., 2010; Johnson et al., 2012), rather than the rhythmic firings we observed in slices. We postulate that patterned activity originating from upstream centers can override the rhythmic activity driven by I_h_ *in vivo*. Alternatively, tonic inhibition *in vivo* may prevent the rhythmic IFs of neonatal neurons, and with the absence of synaptic inputs in slices, rhythmic activity might emerge in the active subset of neurons with high intrinsic excitability, as exemplified by narrow APs and/or elevated spike number in response to current injection (Figure 7). Auditory neurons are particularly enriched in Kv1 and Kv3 channels, with the former being important for suppressing aberrant firings and the latter being critical for rapid repolarization to shorten spike width, enabling high-frequency firing (Brew and Forsythe, 1995; Wang et al., 1998; Ishikawa et al., 2003). Recent experiments suggest that knock-down of Kv3.4 using siRNA results in action potential broadening (Liu et al., 2017). Additionally, Kv1.1 and HCN1 are colocalized in CN (Oertel et al., 2008). It is therefore reasonable to interpret our observations as such that firing and wiring may be reciprocally interlocked processes. Peripheral synaptic innervation may activate the expression of HCN channels in central neurons so as to help initiate their firings, which further boost the postsynaptic excitability by up regulating other voltage-gated ion channels such as Kv1 s and Kv3 s. In return, HCN channel-mediated IFs of central neurons activate unknown retrograde signaling cascade(s) to refine the initial synaptic structure and function, pruning weak synaptic inputs in particular. Genetic silencing of the peripheral inputs and the first-order central neurons coupled with molecular perturbations of activity-dependent retrograde signaling pathways are needed to decipher the causal relationship, if any, between SFs and developmental remodeling in future experiments. Thus far, our results showing BCs with SFs have fewer but stronger synaptic inputs than those without implicate the constructive role of SFs in remodeling early neural circuits in the developing brain.

## Conclusion

In summary, we discovered a form of spontaneous firing activity in neonatal CN neurons that is largely independent of synaptic inputs. Such firings are of intrinsic nature and driven primarily by HCN1 channels which play an important role in promoting synchronized network activity as well as regulating neuron firing patterns and excitability (Kim and Holt, 2013). We propose that intrinsic spiking can serve as important cues for initiating and remodeling synaptic connectivity between peripheral and central neurons in spatially distant nuclei, e.g., IHCs to CNs. Spontaneous activity in peripheral neurons may be instructive, but our findings suggest that coincident spiking in the first central neurons constitutes a fundamental building block in network wiring, potentially enabling early coordinated activity to guide rapid remodeling and maturation of functional circuits in the auditory brainstem and other sensory systems.

## Author Contributions

X-LY, C-YL, H-BS, L-YW and S-KY conceived the project and designed the experiments. X-LY, ML and H-QJ carried out a majority of experiments. H-LP, L-NG, H-WL and Y-ZX participated in pilot experiments and analyzed the data. X-LY, H-BS, C-YL, L-YW and S-KY wrote and revised the article. All authors have approved the final copy of the manuscript and agreed to be accountable for all aspects of the work. All persons designated as authors qualify for authorship, and all those who qualify for authorship are listed.

## Conflict of Interest Statement

The authors declare that the research was conducted in the absence of any commercial or financial relationships that could be construed as a potential conflict of interest.
